# The 10-year trend in drug prescriptions for attention-deficit/hyperactivity disorder (ADHD) in Germany

**DOI:** 10.1007/s00228-020-02948-3

**Published:** 2020-08-17

**Authors:** Thomas Grimmsmann, Wolfgang Himmel

**Affiliations:** 1 German Health Insurance Medical Service (MDK) Mecklenburg-Vorpommern, 19059 Schwerin, Germany; 2 Department of General Practice, University of Göttingen, Humboldtallee 38, 37073 Goettingen, Germany

**Keywords:** Attention-deficit disorder with hyperactivity, Drug prescriptions, Cohort studies, Ambulatory care, Methylphenidate

## Abstract

**Purpose:**

The aim of this study was to analyse whether the global trend in drug prescriptions for attention-deficit hyperactivity disorders (ADHD), as observed during the last years and often criticized as medicalization, have remained stable or shifted.

**Methods:**

This observational study was based on a secondary analysis of data from a large German database including patients with an ADHD diagnosis between 2008 and 2018. Prescription data comprised all important ADHD drugs.

**Results:**

A total of 620 practices delivered data from a total of 77,504 patients (31% of them females) with a diagnosis of AHDH. Nearly 38% (29,396/77,504) of all patients received, at least, one prescription for an ADHS medicine between 2008 and 2018. The number of patients receiving a drug steadily increased annually until 2012 and then slowly fell, but unevenly distributed across the age groups. While the number of younger patients ( ≤ 16 years) receiving a prescription fell by 24% and the defined daily doses (DDDs) remained stable, the number of patients between 17 and 24 years receiving a prescription increased by 113% and the DDDs by 150%. Respectively, the number of older adults (≥ 25 years) with a prescription increased by 355% and the DDDs by 515%. Nearly one-third of older adults received an ADHD medicine only once.

**Conclusion:**

The ever-increasing prescription of ADHD medicines stopped some years ago for children. ADHS and its pharmacological management are increasingly observed among older adolescents and adults, with a different pattern of drug persistence compared with children.

**Electronic supplementary material:**

The online version of this article (10.1007/s00228-020-02948-3) contains supplementary material, which is available to authorized users.

## Introduction

Attention-deficit/hyperactivity disorder (ADHD) is among the most common neurobehavioural problem afflicting children and adolescents. Individuals suffering from this disorder exhibit hyperactivity, inattention, impulsivity and problems in social interaction and academic performance [1]. In a substantial number of cases, the disorder does not remit in puberty but persists into adulthood [2]. Whether late-onset ADHD exists is controversially discussed [3, 4]. The estimated overall population prevalence of ADHD is 7.2% (95% confidence interval: 6.7 to 7.8) [5]; the administrative prevalence of ADHD is 4.33% (95% CI: 4.31–4.34%) for children and adolescents in Germany; for adults, the prevalence is far lower [6, 7].

The number of prescriptions and the number of children who take ADHD drugs have increased dramatically in various European countries [8], and recently, the same trend has been observed in adults as well [7, 9]. If we consider the overall drug prevalence in Germany, there was an increase from 1.3% of children between 5 and 9 years or 2.9% of children between 10 and 14 years in 2005 up to 2.0% or 4.4%, respectively, in the following years [8]. This upward trend seems to have reached a plateau or has been slightly decreasing in recent years for children, while for adults, it is still increasing [7, 10].

There are several concerns about the pharmacological management of ADHD. Methylphenidate, the most commonly prescribed medicine, is associated with an increased risk of non-serious adverse events, such as sleep problems and decreased appetite [11]. Moreover, the widespread use of ADHD medicines, especially the use of methylphenidate in children, has long been criticized as medicalization [12], i.e., the definition and treatment of typical human experiences and emotions as medical conditions under the authority of physicians.

Despite these concerns, new guidelines on the management of ADHD recommend a more liberal use of these drugs. For example, the guideline of the National Institute for Health and Care Excellence (NICE) recommends pharmacotherapy as first-line treatment for adults [13], although research of pharmacotherapy for adults is still largely lacking [2], and methylphenidate is the first-line pharmacological treatment for children over five and adolescents if symptoms are still causing a persistent significant impairment in at least one domain after environmental modifications have been implemented and reviewed. In other words, NICE focuses on the presence of significant impairment in the different domains of everyday life, rather than using the previously used terms of mild, moderate and severe ADHD [13]. Similarly, an updated German guideline (AWMF) [14] recommends these medicines not only for severe cases, as in former versions, but also for moderate cases. These updates were criticized by the public [15] as well as scientists [16] due to a supposed further increase in drug prescriptions. To date, however, we do not know whether the new guidelines led, indeed, to increased drug prescriptions.

Additionally, and in remarkable contrast to these concerns, we witness a constantly repeated warning of an allegedly insufficient awareness of “adult ADHD” [17] and its possible undertreatment [7, 18, 19], accompanied by an increase of administrative prevalence of adult ADHD and a new clinical picture, coined late-onset ADHD [6].

In other words, we face a mixture of fears of overtreatment of children/adolescents and undertreatment of adults. In this situation, new and valid data that highlight the prescription of these drugs are key for optimal treatment and rational discourse. According to Beau-Lejdstrom and Magno Zito [20], it is essential to expand our knowledge of medication use patterns from large observational studies, for example, by measuring the prevalence of ADHD medication use across the age groups and in terms of duration (i.e. persistence of ADHD medication use). The aim of this study was to analyse whether the global trends in ADHD prescriptions observed during the last years have remained stable or shifted, with a special focus on and comparison between children, adolescents and adults.

## Material and methods

### Design

This observational study was based on a secondary analysis of data from a large database provided by IMS/IQVIA [21]. For data preparation, analysis and reporting of the results, we used the RECORD checklist for reporting observational studies [18].

### Database

The IMS® Disease Analyzer contains anonymized data obtained from the practice computers from office-based physicians specializing in various disciplines. The database records, in addition to other data, ICD-10-coded diagnoses, prescriptions, referrals and dates of appointments. The database contains data from 2498 practices [21] and appears to be representative of prescriptions issued by statutory health insurance (SHI)-accredited physicians [22].

The authors obtained data from a part of the database that included all patients with at least one diagnosis of ADHD (ICD-10-CM: F90 attention-deficit hyperactivity disorders) between 2008 and 2018, including information on physician specialty, patient’s age and sex, diagnosis expressed as ICD 10 codes (up to level 4), date of diagnosis and referrals. Prescription data comprised date of visit, product expressed as an anatomical therapeutic chemical (ATC) code, strength and pack size.

All data are physician-related and patient-related only for a single practice so that a patient could not be followed if he or she changed practice.

### Definitions and analysis

#### ADHD drugs

Analysis included drugs approved for treatment of ADHD in Germany: dexamfetamine, lisdexamfetamine and methylphenidate as stimulants and atomoxetine and guanfacine as non-stimulant drugs.

#### Measurement of prescriptions and persistence

Defined daily doses (DDDs) were calculated from the prescribed strength and pack size for each prescription. For persistence, according to the date of the first prescription, we calculated the months between the first and last prescription until there were no further prescriptions within 360 days.

#### Age groups

For some analyses, we divided the patients into 3 age groups as follows: children, adolescents (16 years and younger), young persons (from 17 years to 24 years) and adults (25 years and older), similar to a classification in a French observational cohort study that assessed patterns of methylphenidate use in children and adults [23]. We deliberately deviated from the usual classification of children, adolescents and adults since—compared with our knowledge on children and young adolescents—our knowledge on ADHD medication in the critical phase of development and young adulthood is limited.

#### Statistical analysis

In most instances, the unit of analysis was the ADHD diagnosed patient with a prescribed ADHD medication. Only descriptive statistics, such as absolute and relative frequencies, as well as the means, medians and interquartile ratios (IQRs), were calculated. For a sensitivity analysis, we changed the stop criterion for persistence from 360 days to a gap of up to 180 days.

## Results

### Patients and prescriptions

A total of 620 practices, including 437 primary care physicians, 92 paediatricians, 64 neurologists and 27 psychiatrists, delivered data from a total of 77,504 patients with a diagnosis of ADHD. The patients were, on average, 18.9 (median: 12) years old at the time of the first diagnosis of ADHD and/or the first ADHD prescription; 30.8% of them were female.

Nearly 38% (29,396/77,504) of all patients received at least one prescription for an ADHD medicine during the time period under study; 26% (7704 / 29,396) of them were female.

**Fig. 1:**
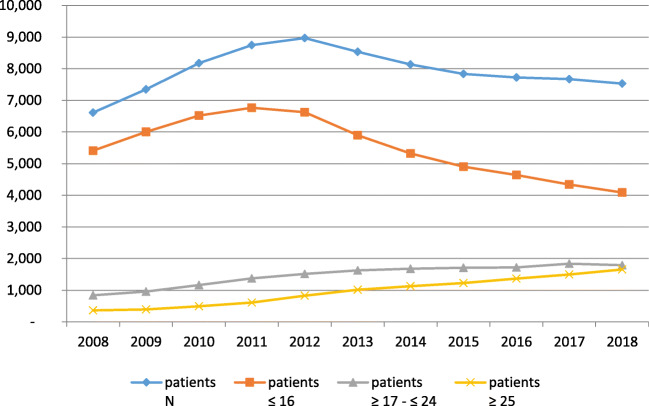
Patients with an ADHD prescription; according to different age groups, 2008 to 2018

The number of patients receiving a drug steadily increased annually from 6613 in 2008 to 8969 in 2012 and then fell to 7533 in 2018 (Fig. 1), with a highly unequal distribution between children/adolescents on the one side and adults on the other side. The number of younger persons (≤ 16 years) receiving ADHD medicine continuously fell from 6767 in 2011 to 4085 in 2018, and the number of older persons (≥ 17 years) continuously rose throughout the ten years under study (Fig. 1). In those ≥ 17 and ≤ 24 years, there was an increase of more than 100% (from 839 persons in 2008 to 1791 in 2018); in those ≥ 25 years, there was an increase of more than 350% (from 364 persons in 2008 to 1657 in 2018). While these two groups together represented 18% of all persons receiving ADHD medicine in 2008, they represented 46% in 2018.

**Fig. 2:**
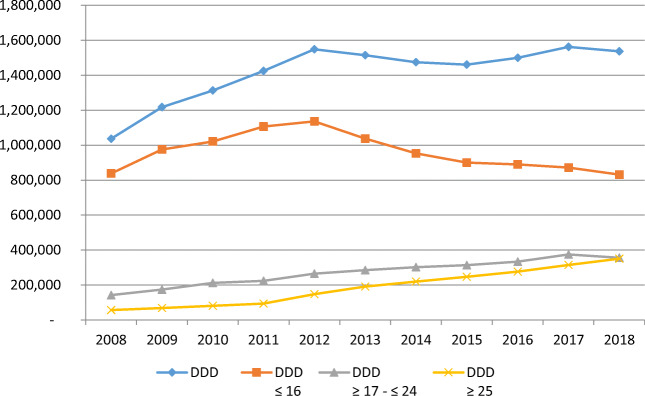
Prescribed ADHD medicines for patients with a diagnosis of ADHD, in DDDs; 2008 to 2018

The situation is similar if we look at the quantity of medicines. The quantity rose from 1,037,210 DDDs in 2008 to 1,537,449 DDDs in 2018, an increase of 48%, while the number of patients increased by only 14% (Fig. 2). Consequently, the increase in the number of DDDs was due to a continuous increase in the average number of DDDs per patient from 157 DDDs/patient/year in 2008 to 204 DDDs in 2018, with a somewhat stronger increase in older adults than in the remainder of the studied population (see Table; Appendix A). Like the number of patients, the total number of DDDs for younger persons decreased after 2012 but only slightly and remained stable for the following years. For older adults (≥ 25 years), we saw an increase of more than 500% in the number of DDDs, from 57,029 in 2008 to 350,656 in 2018 (Fig. 2), while the number for those ≥ 17 and ≤ 24 years more than doubled (from 141,975 to 355,486).

Methylphenidate (Ritalin) is still by far the most prescribed medicine for ADHD, followed by lisdexamfetamine, which was first prescribed in 2013, and atomoxetine. The three substances differ in regard to the targeted population. Methylphenidate was the dominant substance 10 years ago, with more than 90% of patients receiving this drug. This number decreased for children/adolescents from 92% to 81% of the patients (or, in terms of DDD, from 93% to 70%) and from 98% to 91% for adults (from 99% to 91% DDDs). Lisdexamfetamine increased, especially in children/adolescents (2018: 21% patients; 23% DDD). The share of atomoxetine grew, but not steadily, in adults and decreased in children/adolescents.

**Fig. 3:**
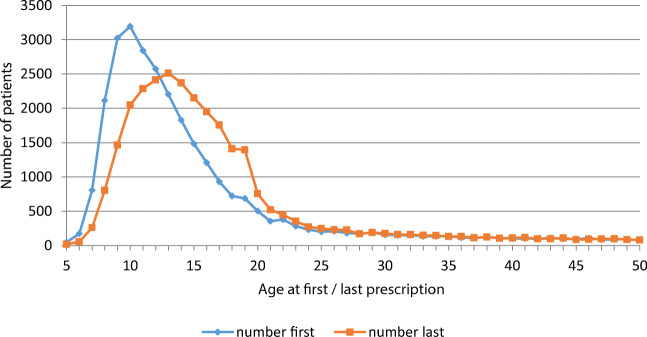
Number of patients, according to the patient’s age at therapy initiation (first prescription) and last prescription

When we considered the patient’s age at therapy initiation, we observed some age differences (Fig. 3). During the study period, drug therapy was very frequently initiated mostly in younger persons (≤ 16 years), with a peak at the age of 10 years, followed by a more or less linear decrease in the age of first prescription until the age of 21. This finding corresponds to the age of the “last prescription”, i.e., the point of time when no further prescription was issued. The group of children aged 13 showed the highest number of patients receiving a “last prescription”, which then steadily decreased until the age of 25.

### Persistence

**Fig. 4:**
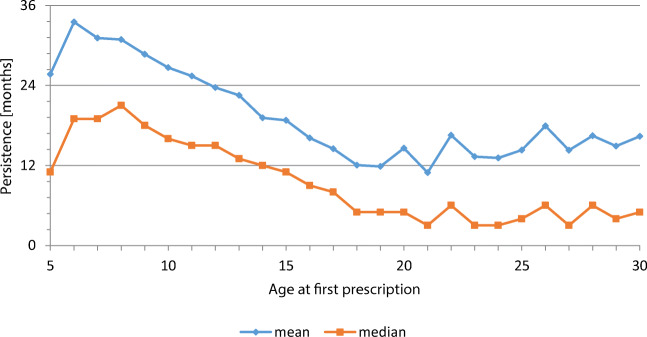
Drug persistence, according to age at first prescription

Figure 4 shows how long patients received a prescription for a medication, calculating the months between the first and last prescription. This analysis is based on those patients who received a prescription (*n* = 29,396). These patients received their prescriptions of an ADHD medicine for 22 months, on average (median: 12; first quartile: 34; third quartile: 2). The results differed substantially according to the age of the patients. While especially the patients younger than 12 years remained under treatment, on average, for 2 years or longer, adult patients received medication for a much shorter time (Fig. 3). A high percentage (27%) of older adults, so-called spot users, received an ADHD medicine only once, compared with 17% of spot users among children.

The sensitivity analysis revealed similar trends, but inevitably, with a generally lower level of persistence in the case of the 180 d-criterion (see Figure; Appendix B).

### Comorbidities and drug prescriptions

Approximately two-thirds (52,319/77,504) of all patients had at least one other diagnosis from Chapter 5 ICD-10-CM (mental, behavioural and neurodevelopmental disorders; F 10–F 99). This figure was somewhat higher in children/adolescents (76%) than in those aged 17 to 24 years (60%) or ≥ 25 years (63%). More importantly, the single diagnoses differed markedly between the age groups (Table 1). Not surprisingly, the younger patients were more often diagnosed as having “pervasive and specific developmental disorders” (54%), especially speech and language disorders, while these diagnoses were not common in adults. Vice versa, one-third of the adult patients were diagnosed with “mood (affective) disorders” (F30–F39), whereas nearly none of the children received this diagnosis. “Mental disorders” (F40–F48) were more frequent in adults, but “reaction to severe stress, and adjustment disorders” (F43) was identified in approximately 10% of the patients in all age groups.

**Table 1:** Psychosomatic/psychiatric comorbidity and ADHD drugs

	Children (≤ 16 years)			Adolescents, younger adults (17–24 years)			Older adults (≥ 25 years)		
			ADHD drugs			ADHD drugs			ADHD drugs
Psychiatric/psychosomatic diagnoses	N	(%)	%	N	(%)	%	N	(%)	%
Only F90 (ADHD)	8071	(24.3)	28.4	9887	(39.6)	51.0	7216	(37.3)	32.9
Additional F-diagnoses	25,121	(75.7)	31.3	15,042	(60.4)	53.6	12,124	(62.7)	31.0
All	33,192	(100)	30.6	24,929	(100)	52.6	19,340	(100)	31.7
Additional F-diagnoses									
Mental disorders due to known physiological conditions (F01–F09)	208	(0.6)	31.3	175	(0.7)	45.7	1624	(8.4)	5.6
Mental and behavioural disorders due to psychoactive substance use (F10–F19)	105	(0.3)	30.5	670	(2.7)	53.0	2479	(12.8)	25.4
Schizophrenia, schizotypal, delusional, and other non-mood psychotic disorders (F20–F29)	45	(0.1)	48.9	189	(0.8)	47.6	554	(2.9)	37.7
Mood [affective] disorders (F30–F39)	1064	(3.2)	38.7	2202	(8.8)	56.4	6308	(32.6)	33.7
Anxiety, dissociative, stress-related, somatoform and other nonpsychotic mental disorders (F40–F48)	5939	(17.9)	33.0	4645	(18.6)	51.6	6588	(34.1)	24.1
Behavioural syndromes associated with physiological disturbances and physical factors (F50–F59)	1162	(3.5)	25.7	598	(2.4)	50.5	911	(4.7)	24.5
Disorders of adult personality and behaviour (F60–F69)	2524	(7.6)	26.0	1779	(7.1)	51.4	1706	(8.8)	35.1
Intellectual disabilities (F70–F79)	1497	(4.5)	29.5	792	(3.2)	46.8	276	(1.4)	30.8
Pervasive and specific developmental disorders (F80–F89)	17,778	(53.6)	29.3	6854	(27.5)	56.1	625	(3.2)	56.0
Behavioural and emotional disorders with onset usually occurring in childhood and adolescence (F91–F98)	14,252	(42.9)	33.5	7248	(29.1)	55.2	1467	(7.6)	61.2
Unspecified mental disorder (F99)	359	(1.1)	19.8	202	(0.8)	53.5	119	(0.6)	26.9

Interestingly, these comorbidities played only a minor role in a doctor’s decision regarding whether to prescribe an ADHD drug for patients with a diagnosis of ADHD. While 38.6% of the persons with only an ADHD (F90) diagnosis and no other diagnosis from ICD-Chapter 5 ICD-10-CM received a drug, it was 37.6% of those with at least one additional F-diagnosis (Table 1). Even when a patient had more than 2, 3 or 4 psychiatric diagnoses, the likelihood of receiving an ADHD drug did not increase; quite the opposite, the rate decreased slightly from 40% of patients receiving a drug if they had one additional psychiatric diagnoses to 34% of those with four additional psychiatric diagnoses. While the rate of patients with a prescription was higher in adolescents, it made no difference whether the patients had an additional psychiatric diagnosis or not.

## Discussion

### Summary of the main findings

The overall prescription of medicines to treat ADHD in Germany remained stable over the last few years or increased only slightly after new guidelines worldwide recommended a more liberal use of these medicines. ADHD and its pharmacological management are increasingly observed among older adolescents and adults. Older adults (≥ 25 years) use ADHD medicines, on average, for a much shorter period of time than children and adolescents. Psychiatric comorbidities were frequent among persons with an ADHD diagnosis but did not seem to be decisive for a drug prescription.

### Strengths and limitations of the study

We had access to a large and continuous data set from community practices, covering general practices as well as paediatric, neurological and psychiatric practices in Germany. The data comprised the last 10 years, with diagnoses and drug prescriptions as well as the age and sex for persons with an ADHD diagnosis. The sample of practices was deemed to be representative of German practices [22] and was evenly distributed throughout the country.

A limitation of the data source is that it only documents prescribed medications but not whether they are really dispensed from a pharmacy. However, comparing our main results with global prescription data from German health insurance companies, our study approach seemed to yield valid results. Lohse and Müller-Oerlinghausen [24, 25], for example, reported a sharp increase in methylphenidate from 13 million DDDs in 2000 to 58 million DDDs in 2012, with a slight reduction to 53 million DDDs in 2018; however, this decrease was compensated by an increase in lisdexamfetamine from approximately 1 million DDDs when first released in 2013 to 8 or 10 million DDDs in the last 3 years. These findings are exactly in line with our results.

We could follow up the participants using a pseudonymized code but only within one practice and not across several practices. We probably underestimated, to a certain degree, drug supply and persistence of those who received their prescriptions from more than one practice or changed the practice during the time period under study. Patients, however, do not change practice frequently in Germany, with more than 85% of the patients visiting only the same, or maximum two general practitioners (GPs) and more than 90% visiting only the same or a maximum of 2 paediatricians, neurologists or psychiatrists [26]. However, we could not follow up patients when changing the practice and the same patients may consult several practices of the IQVIA practice panel. So, it was not possible to define the population at risk and we abstained from calculating the disease prevalence for the different age groups.

### Comparison with the literature and meaning of the results

The frequency and quantity of prescriptions of methylphenidate are still a major concern in the scientific and public communities (not only) in Germany [27]. Portions of the public and some scientists fear that new ADHD guidelines will lead to a further increase in these drugs [16]. As far as children are concerned, our study did not detect any indications for such a trend to date. Prescriptions for children are still on a high level, compared with the levels two decades ago.

The cessation or slight decrease in methylphenidate use in recent years seen in our study was also observed in another German pharmacoepidemiological study [28]. According to the authors of this study, this trend could be caused by prescription restrictions by the German regulatory agency, a more cautious stance towards pharmacotherapy and medicalization among physicians and parents, and warnings regarding the potential cardiovascular risks of methylphenidate medications. However, this latter argument is only partially convincing as a counter-argument against the medicalization hypothesis since the authors did not consider compensation by other ADHD medicines in youth.

Moreover, and in remarkable contrast to these concerns, we witnessed a constantly repeated warning of an allegedly insufficient awareness of “adult ADHD”, including an ongoing debate whether a significant proportion of adults remain undiagnosed and, consequently, untreated [7, 17–19]. The results and trends observed in our study can be condensed to three arguments that may inform this debate.

First, many studies have documented an increase in adults diagnosed with ADHD who are receiving respective medicines worldwide, with relative increases in medication use per year of 17 to 19% for adults until 2015, compared with only 9 to 15% for non-adults [29]. Two US studies also reported that the proportion of adults treated with stimulants grew rapidly from 1999 to 2010 and again from 2010 to 2014, in contrast to youths, who had a modest increase in stimulant use [30, 31]. Extending the study period until 2018, we could show that this trend is still active in Germany, but only for adults and older adolescents who now represent nearly half of the ADHD population who receive medication. Therefore, pharmacotherapy for adults has developed greatly over the last decade, to an extent that compensated for the slight decline in ADHD medicines in children. This development does not necessarily exclude the possibility that a considerable part of adults are still undertreated but we witness increased attention towards adults and increased willingness to prescribe them ADHD medicines.

Second, based on an estimated transition rate from childhood to adult ADHD of approximately 50% [32], several authors proposed that a suboptimal transition from child to adult services or adult mental health services and a reluctance to consider ADHD as a disease of later years and later onset may lead to an undertreatment of adults with symptoms of an ADHD [33, 34]. However, there was no particular discontinuation of treatment during the transition from adolescence to early adulthood (Fig. 3). We could not detect a specific high number of patients receiving their “last prescription” at the age of 16, 17 or 18 years; rather, the rate of “last prescriptions” continuously declined from the age of 13 years. While older age was a predictive factor for lower persistence in our study as in a Taiwan study [35], we could not detect a specific low persistence in the transition group so that it is rather unlikely that the transition phase is a trigger for the discontinuation of treatment.

Third, recent research [4] suggests that the emergence of ADHD symptoms in later years may not originate from ADHD itself but rather reflects one or more other mental health disorders with symptoms similar to ADHD as follows: depression is associated with difficulty focusing on tasks, anxiety may cause distractibility, substance use may lead to low arousal levels and lack of motivation, and so on. This concept may help to explain why so many adults (27%) in our sample were spot users, i.e., they did not receive or demand a second prescription after an initial therapy with an ADHD medicine. Although we do not know who, the patient or the doctor, stopped pharmacotherapy after an initial trial of an ADHD drug for a rather short time, this lack of persistence may indicate that one party or both parties tried a sort of “empirical” therapy in the case of symptoms similar to ADHD symptoms—but often stopped them if not successful after a first drug trial. One could assume a higher rate of adults experiencing intolerable adverse effects, or incompatibility with their drugs for other medical problems, e.g., cardiac drugs. Indeed, a meta-analysis [36] found that adult ADHD patients randomized to CNS stimulant treatment demonstrated a statistically significant increased resting heart rate and systolic blood pressure findings compared with subjects randomized to placebo. However, none of the studies reported any serious cardiovascular events nor was the rate of drug discontinuation due to cardiovascular symptoms different between the treatment and placebo groups.

The obvious sensitivity towards such ADHD symptoms together with therapeutic attempts also contradicts the hypothesis that doctors neglect adult ADHD. Although some adults may still be undertreated or do not receive medicines at all, the lack of persistence observed in our study may be, at least partly, explained by “overtreatment” of those adults who were treated empirically with ADHD medicines for symptoms similar to ADHD symptoms but stopped the treatment probably because they did not experience a benefit. Different patterns of methylphenidate use among adults were also observed in Pauly et al.’s study where drug persistence decreased with age and adults who used methylphenidate had more psychiatric disorders than the younger ones [23].

## Conclusion

The ever-increasing prescription of ADHD medicines, a major concern of the public and many researchers, stopped some years ago and even decreased in some age groups. While new guidelines obviously did not lead to an increasing trend in methylphenidate use among children, we should not overrate the decline in the use of these medicines. The reduction was very small, so the use of methylphenidate is still high.

However, there are still not enough data about the benefits and risks of long-term therapy, and post-marketing surveillance studies in community populations may be helpful. Even so, a stronger focus on the role of comedication—particularly interacting comedication or medication classes like atypical antipsychotics possibly prescribed for ADHD—may be helpful to better understand the decision to prescribe or not to prescribe an ADHD medicine and drug persistence.

What is surprising is the call to give adults and their pharmacological supply more attention. Considering the strong rise in drug supply of adults and the notable prescriptions pattern for this patient group, our analysis shows that doctors pay greater attention towards adults with a possible diagnosis of ADHD and a possible undertreatment of them.

Our pharmacoepidemiological analysis does not allow for inferences about the adequacy of the drug trends or the benefits of the medicines prescribed. At best, this analysis deepens our knowledge about recent trends and indicates risks and blind spots [2] in the scientific and public debate about the use of psychiatric medications for ADHD.

## Electronic supplementary material

ESM 1 (DOCX 14.6 kb).

ESM 2 (DOCX 16.5 kb).

## Data Availability

The data that support the findings of this study are available from third party (data owners). Restrictions apply to the availability of these data, which were used under licence for this study. Data are available from the authors with the permission of third party.
