# Supplementary material for: The 10-year trend in drug prescriptions for attention-deficit/hyperactivity disorder (ADHD) in Germany
Source: Eur J Clin Pharmacol. 2020 Aug 17;77(1):107–15. doi: 10.1007/s00228-020-02948-3 (PMC7782395; doi:10.1007/s00228-020-02948-3)
Supplement: Supplementary file 1 — (DOCX 14.6 kb). [file 228_2020_2948_MOESM1_ESM.docx]

**Appendix A**

**Table** DDDs per patient, according to age groups, 2008 to 2018

| Year | DDDs /patients N | DDDs / patients ≤ 16 | DDDs /patients ≥ 17 - ≤ 24 | DDDs / patients ≥ 25 |
| --- | --- | --- | --- | --- |
| 2008 | 157 | 155 | 169 | 157 |
| 2009 | 166 | 163 | 181 | 173 |
| 2010 | 161 | 157 | 182 | 162 |
| 2011 | 163 | 164 | 163 | 155 |
| 2012 | 173 | 171 | 175 | 178 |
| 2013 | 177 | 176 | 175 | 188 |
| 2014 | 181 | 179 | 180 | 194 |
| 2015 | 186 | 184 | 184 | 201 |
| 2016 | 194 | 192 | 194 | 202 |
| 2017 | 204 | 201 | 204 | 211 |
| 2018 | 204 | 204 | 198 | 212 |
