# Supplementary material for: The 10-year trend in drug prescriptions for attention-deficit/hyperactivity disorder (ADHD) in Germany
Source: Eur J Clin Pharmacol. 2020 Aug 17;77(1):107–15. doi: 10.1007/s00228-020-02948-3 (PMC7782395; doi:10.1007/s00228-020-02948-3)
Supplement: Supplementary file 2 — (DOCX 16.5 kb). [file 228_2020_2948_MOESM2_ESM.docx]

**Appendix B**

Figure Drug persistence, according to age at first prescription (sensitivity analysis with a gap of up to 180 days as criterion for persistence)
